# Bio-Functional and Structural Properties of Pasta Enriched with a Debranning Fraction from Purple Wheat

**DOI:** 10.3390/foods9020163

**Published:** 2020-02-08

**Authors:** Parisa Abbasi Parizad, Mauro Marengo, Francesco Bonomi, Alessio Scarafoni, Cristina Cecchini, Maria Ambrogina Pagani, Alessandra Marti, Stefania Iametti

**Affiliations:** 1 Department of Food, Environmental, and Nutritional Sciences (DeFENS), Università degli Studi di Milano, Via G. Celoria 2, 20133 Milan, Italy; parisa.abbasi@unimi.it (P.A.P.); mauro.marengo@unito.it (M.M.); francesco.bonomi@unimi.it (F.B.); alessio.scarafoni@unimi.it (A.S.); ambrogina.pagani@unimi.it (M.A.P.); 2 Consiglio per la Ricerca in Agricoltura e L’analisi Dell’economia Agraria (CREA), Centro di Ricerca Ingegneria e Trasformazioni Agroalimentari, Via Manziana 30, 00189 Roma, Italy; cristina.cecchini@crea.gov.it

**Keywords:** pigmented wheat, anthocyanins, polyphenols, alpha-amylase inhibition, anti-inflammatory activity

## Abstract

A colored and fiber-rich fraction from the debranning of purple wheat was incorporated at 25% into semolina- and flour-based pasta produced on a pilot-plant scale, with the aim of increasing anthocyanin and total phenolic content with respect to pasta obtained from whole pigmented grains. The debranning fraction impaired the formation of disulfide-stabilized protein networks in semolina-based systems. Recovery of phenolics was impaired by the pasta making process, and cooking decreased the phenolic content in both enriched samples. Cooking-related losses in anthocyanins and total phenolics were similar, but anthocyanins in the cooked semolina-based pasta were around 20% of what was expected from the formulation. HPLC (High Performance Liquid Chromatography) profiling of phenolics was carried out on extracts from either type of enriched pasta both before and after cooking and indicate possible preferential retention of specific compounds in each type of enriched pasta. Extracts from cooked samples of either enriched pasta were tested as inhibitors of enzymes involved in glucose metabolism and uptake, as well as for their capacity of suppressing the response to inflammatory stimuli. Results of both biological tests indicate that the phenolics in extracts from both cooked pasta samples had inhibitory capacities higher than extracts of the original debranning fraction at identical concentrations of total bioactives.

## 1. Introduction

Many studies have shown that anthocyanins and other polyphenolic compounds have anti-inflammatory and antioxidant properties that may play a positive effect in preventing chronic diseases ranging from cardiovascular diseases to metabolic syndrome. The relationship between specific anthocyanins and their biological activities, such as anti-inflammatory, anti-obesity, anti-diabetes, has been the subject of a number of recent reviews [1,2]. Anthocyanins and polyphenols have also been reported to control intracellular signaling cascades as the process of inflammation progresses within the cells [3,4,5]. Numerous studies have demonstrated that anthocyanins can exert the beneficial effects in diabetes by acting on various molecular targets and regulate different signaling pathways in multiple organs and tissues such as liver, pancreas, kidney, adipose, skeletal muscle, and brain [6].

A critical aspect of most of the reported bioactivities for this class of chemical species relates to the poor absorption of several phenolics that impairs their presence at high enough concentration in biological fluids [7,8]. However, many of the health benefits associated with anthocyanins bioactivity relate to the effects these molecules reportedly exert on proteins in the intestine. Among the significant targets that do not require a transit of phenolics across the gastrointestinal epithelia are enzymes involved in glucose metabolism, such as pancreatic amylases involved in the starch enzymatic breakdown and the brush-border alpha-glucosidase relevant to glucose uptake [9,10,11,12,13,14].

One possible strategy to ensure adequate uptake of anthocyanins and phenolics is their incorporation into staple foods [8,15]. In this frame, pigmented grains have received particular attention as they may represent the starting ingredients to produce staple foods such as pasta or bread. High concentrations of phenolic compounds are present in the outer layers of a number of varieties of common grains such as wheat, corn, and rice [16]. In the case of colored grains, anthocyanins are responsible for their purple, blue, or red color [17]. These compounds are present in various amounts in bran layers as either the glycosylated or aglycone form [18].

The growing interest for new products and the visual appeal of naturally colorful products has led to the introduction of “colored” bread and pasta to the market. Most of the available products are prepared by using whole grains as the source of anthocyanins [16,19]. Recently, an innovative milling process has been developed in order to separate bran components to be used in specific processes [20,21]. In the case of colored grains, the usually discarded outermost part of the kernels is a suitable ingredient for the production of enriched food. According to Zanoletti et al., [20], it was possible to produce pasta with a high amount (up to 15% (*w*/*w*)) of bran fraction, with fiber and polyphenol content sensibly higher than in products prepared from the corresponding whole grains. The enriched pasta samples were characterized mainly in terms of chemical composition and cooking behavior, and only limited data are available for the retention of the bioactive properties of the incorporated materials.

This study attempts to fill evident gaps in the studies mentioned above by studying the effects of processing (and cooking) on retention of the bioactives’ functionality in pasta samples prepared by incorporating at least 25% of anthocyanin-rich bran fractions from purple wheat debranning into pasta made from semolina or common wheat flour. This amount was chosen to provide nutritionally relevant amounts of both dietary fibers and phenolics in a product with attractive color features, avoiding in the meantime excessive changes in the pasta-making process and allowing the retention of the structural integrity of the cooked product. The focus in this study was on the in vitro inhibitory capacity of phenolics toward specific enzymes involved in the carbohydrate metabolism, as well as on their anti-inflammatory properties.

## 2. Materials and Methods

### 2.1. Chemicals and Enzymes

Unless otherwise specified, all chemicals and enzymes (namely, rat intestinal acetone powders of alpha-glucosidase (EC 3.2.1.20) and porcine pancreatic alpha-amylase (EC 3.2.1.1)) were from Sigma-Aldrich (Milan, Italy).

### 2.2. Raw Materials

An anthocyanin-rich bran fraction (henceforth, DF) was obtained by the debranning of commercial purple wheat, essentially as reported in [20]. The DF used in the studies reported below corresponds to an abrasion level of 3.7%, with respect to the whole grain and had a particle size in the 500–700 µm range. Refined common wheat flour (12.5% protein, dw; ashes <0.5%, dw) and durum wheat semolina (14.4% protein, dw; ashes 0.85%, dw) were provided by Molino Quaglia (Vighizzolo d’Este, Padua, Italy) and by F.lli De Cecco (Fara San Martino, Chieti, Italy), respectively.

### 2.3. Pasta

Pasta was produced from either commercial flour or semolina that was enriched by dry mixing with 25% (*w*/*w*) of the bran fraction obtained by the debranning of purple wheat (DF, see above). Macaroni-shaped pasta samples were produced using the DeFENS pilot plant, essentially as described in [22]. In particular, moisture in the dough was adjusted to a final level of 31.8%, and mixing and extrusion were completed in 20 min. Drying was carried out at 60 °C for 12 h. Each sample of pasta was cooked in tap water (1 L water per 100 g pasta) at the optimum cooking time, according to the AACC method 16–50 [23]. The sensory acceptability of the cooked pasta was assessed by ten untrained panelists. Prior to further characterization, samples of dried uncooked pasta were ground to <250 µm in a laboratory mill, whereas samples of cooked pasta were frozen in a deep freezer at −80 °C and lyophilized (Alpha 2-4 LD freeze dryer, Martin Christ, Osterhode am Harz, Germany) prior to further characterization.

### 2.4. Protein Solubility and Thiol Accessibility

The solubility of proteins in pasta samples was determined in triplicate using buffers of increasing dissociating ability, as described elsewhere [24,25]. Results are expressed as (mg soluble protein)/(g total proteins) to account for the protein content of individual samples, assessed through a dye-binding method [26]. Accessible thiol groups were determined (in triplicate) by using the spectrophotometric thiol reagent 5,5′-dithiobis-(2-nitrobenzoate) (DTNB) as also described in [24,25]. Results are expressed as μmol thiols/g total protein, to account for the protein content of individual samples.

### 2.5. Phenolics Extraction

Each sample (2 g) was defatted with petroleum ether and extracted twice with 15 mL of an ethanol/HCl mixture (15 mL of a mixture made up of 65 volumes of 95% ethanol and 35 volumes of aqueous 0.3 M HCl). The pooled ethanol/HCl extracts were used for analytical measurements and HPLC profiling. Extracts to be used with Caco-2 cells were by using water in place of dilute HCl, in order to avoid interference with the cellular assays and cell viability issues [13]. Assays were carried out in triplicate for each sample.

### 2.6. Determination of the Total Polyphenols and the Total Anthocyanins Content

Total polyphenols (as gallic acid equivalents) and total anthocyanins (as cyanidin-3-*O*-glucoside) were measured as in [27] on individual extracts from both cooked and uncooked pasta samples.

### 2.7. Anthocyanin and Phenolics Profiling by RP-HPLC

RP-HPLC profiling was performed on a C18 column (5 μm, 4.6 mm × 250 mm, Waters, Milan, IT) fitted to a Waters 600 E HPLC, equipped with a 996 PDA (Photo Diode Array) detector, by adapting procedures reported elsewhere [9]. Extracts (0.1–0.2 mL) were loaded on the column, and components eluted at a solvent flow of 0.8 mL min^−1^, using a gradient from 100% A (0.1% trifluoroacetic acid in water) to 100% B (0.1% trifluoracetic acid in acetonitrile): 0% to 5% B in 5 min; 5% to 40% B from 5 to 40 min; 40% to 70% B from 40 to 48 min. The eluate was monitored at 520 nm (for anthocyanins), 350 nm (for rutin and quercetin), 320 nm (for ferulic acid), and 280 nm (for catechin and epicatechin). Calibration was carried out by using suitable standards, and results are expressed as content of individual species in the original sample, on a dry matter basis.

### 2.8. Enzyme Inhibition Studies

Alpha-Glucosidase: Rat intestinal acetone powder was used in these assays, following established procedures [10,11,12,13,14] and testing inhibition by ethanol/HCl extracts from individual samples, diluted as appropriate in ethanol/HCl. Blanks were prepared in the absence of enzyme and of the ethanol/HCl mixture, whereas controls were complete reaction mixtures containing the appropriate volumes of the ethanol/HCl mixture used for extraction, but no bioactives. Acarbose (from a stock solution in ethanol/HCl) was used as the reference inhibitor. Tests were carried out in triplicate.

α-Amylase: Enzymatic activity was measured according to established procedures [10,11,12,13,14], and inhibition was tested by using ethanol/HCl extracts from individual samples, diluted as appropriate in ethanol/HCl. Blanks were prepared in the absence of enzymes, whereas controls were complete reaction mixtures, containing the appropriate volumes of the ethanol/HCl mixture used for extraction, but no bioactives. Acarbose (from a stock solution in ethanol/HCl) was used as the reference inhibitor. Tests were carried out in triplicate.

### 2.9. Immunomodulatory Properties of Extracts

Human intestinal epithelial Caco-2 cells were provided by Maria Rosa Lovati (Dipartimento di Scienze Farmacologiche e BioMolecolari, University of Milan, Milan, Italy). All experiments involving cultured Caco-2 cells have been carried out in the DeFENS Cell Culture Laboratory, a core facility of the Department of Food, Environmental and Nutritional Sciences (University of Milan, Milan, Italy). The experimental setup allows us to monitor the effects of extracts for inhibiting IL1β-stimulated synthesis of NF-κB in the transfected Caco-2 cells. In short, the test measures inhibition of luciferase expression in Caco-2 cells transiently transfected with the pNiFty2-Luc plasmid (InvivoGen, Rho, Italy), that combines five NF-κB binding sites with the luciferase reporter gene *luc*, allowing expression of the luciferase gene in the presence of NF-κB upon stimulation with interleukin 1β (IL1β) at a final concentration of 20 ng mL^−1^ in the presence/absence of extracts from individual samples. It should be noted that the extracts used in this study were prepared with aqueous ethanol in the absence of HCl, as noted above (Section 2.5). The experimental setup for these experiments has been reported in detail elsewhere [13]. The activity of the expressed luciferase was measured in cellular extracts by adding ATP and D-luciferin, followed by monitoring bioluminescence in a VICTOR3 1420 Multilabel Counter (PerkinElmer, Waltham, MA, USA) Each assay was carried out at least in triplicate.

### 2.10. Statistical Analysis

Analysis of variance (one-way ANOVA) was carried out by using Statgraphic Plus v. 5.1 (StatPoint Inc., Warrenton, VA, USA). The addition of DF to each pasta samples was considered as a factor for ANOVA. Results are reported as averages ± SD. The number of replicates for individual measurements is given under the Materials and Methods section for individual measurements, and in the legend to individual tables.

## 3. Results and Discussion

### 3.1. Molecular Organization of Proteins in Enriched Pasta

The possible effects of incorporating high levels of a bran-derived fraction into pasta on the overall structure of the protein network in the product were investigated by taking into account the conditional solubility of proteins and the accessibility of cysteine thiols to water-soluble reagents in the uncooked products [25,28]. The conditional solubility approach offers a simple way for estimating the nature of interprotein bonds, due to their different sensitivity to the action of chaotropes in the presence/absence of disulfide-breaking agents. Measuring the accessibility of residual thiols in the presence/absence of chaotropes provides information on the overall compactness of the protein network and complements protein solubility data.

Results are presented in Table 1, which also include values of these parameters for reference semolina-only pasta made in the same pilot plant under very similar processing conditions. The protein solubility data in Table 1 indicate the prevalence of urea-sensitive hydrophobic interactions in protein networks formed from wheat flour, as well as their lower compactness with respect to those formed by proteins in semolina. However, a comparison with the reference semolina pasta highlights the well-known “destructive” effect of the addition of a bran-derived fraction on the formation of a disulfide-stabilized protein network in semolina-based systems [29]. This is evident when considering that the DTT-dependent increase in soluble proteins is about 25% in the enriched pasta, whereas, in the reference semolina-based pasta, the breakdown of disulfide bonds by DTT results in a 4-fold increase of the amount of solubilized proteins.

A comparison of data on thiol accessibility is also informative, as the number of accessible thiols in either enriched pasta sample is by far lower than what expected from the properties of the starting material or—in the case of semolina-based enriched pasta—from the figures obtained for non-enriched samples. Freely accessible thiols (i.e., those accessible in the absence of chaotropes) in wheat flour or semolina proteins are in the 3–4 micromolar range when expressed on a protein basis [24,25]. The addition of chaotropes results in a 3-fold increase of accessible thiols for wheat flour-based pasta, and almost doubles the amount of accessible thiols in semolina.

A detailed molecular-level investigation of these observations is beyond the scope of this report. However, it seems reasonable to hypothesize that the observed decrease in accessible thiols—regardless of the presence/absence of chaotropes—may relate to some physical features of the enriched pasta that do not necessarily involve the properties of the protein network itself and may involve protein/polysaccharide interactions, as observed, for instance, in a rice-based pasta [30].

### 3.2. Total Anthocyanins and Total Polyphenol Incorporation in Enriched Pasta

The total anthocyanins content (TAC) and the total polyphenol content (TPC) in both cooked and uncooked pasta samples—prepared from either common wheat flour or semolina and 25% (*w*/*w*) of the polyphenol-rich fraction obtained from the debranning of purple wheat grains (DF)—are reported in Table 2. The values are compared with those expected from the content of each subclass of phenolics in the original bran fraction used to enrich both pasta samples (TAC, 690 mg Cya-3-O-glycoside eq/kg; TPC, 47,400 mg GAE/kg [13,20]). Separate analyses indicated that the contribution of phenolics from either the wheat flour or the semolina used in this study was negligible.

The data in Table 2 indicate an apparent decrease in both TAC and TPC in uncooked pasta samples with respect to what expected from the mixing formula. Losses in either family of phenolics may have occurred in the pasta-making process (most likely as a consequence of the drying steps in pasta production [7,31,32]), but the observed decrease may also be attributed to difficulties in recovering either species from the highly structured protein matrix formed upon kneading and drying.

The fact that apparent recovery figures in uncooked pasta are lower for semolina-based samples than for the flour-based ones offers circumstantial support for the latter hypothesis. In other words, the stiff protein network likely present in pasta could make the ethanol/HCl extraction procedure less effective, an effect most evident in semolina-based pasta because of the high protein content of semolina and the relevance of covalent disulfide bonds in the stabilization of interprotein networks formed by *T. durum* (*Triticum durum*) proteins in semolina-based dough in contrast with the prevalence of non-covalent hydrophobic interactions in the stabilization of interprotein networks formed by *T. aestivum* proteins in wheat flour dough [33,34].

As also evident from Table 2, cooking induced a further decrease of the phenolics content in both pasta samples. At this stage, we are unable to discriminate between this decrease being due to the release of the bioactives in the cooking water or to their sensitivity to temperature. In general, it seems safe to assume that there should be no issues for the yield of the ethanol/HCl extraction procedure when applied to cooked and lyophilized pasta samples, given the highly porous structure of the lyophilized samples.

It may be noted that the losses observed upon cooking flour-based enriched pasta are similar when considering anthocyanins alone or total phenolics. Conversely, cooking semolina-based enriched pasta gave loss in total phenolics slightly lower than those observed in common wheat-flour enriched pasta, as expected from the higher tenacity of the protein network in semolina-based pasta according to plentiful literature reports and to the protein network stability data reported in the previous section of this report. In spite of this, losses in anthocyanins upon cooking semolina-based enriched pasta were the highest, resulting in the final content of these species not exceeding 22% of what was calculated for the untreated formulation, based on proportion among individual ingredients and on their content in the various bioactives.

HPLC profiling was used to address whether any particular chemical species was involved in the compositional changes reported in Table 2 before and after cooking. To avoid possible sources of confusion [9], the HPLC profiling in this study takes into account only the aglycones of the most abundant species present in ethanol/HCl extracts of DF, namely, cyanidin and delphinidin (as representative of anthocyanins), and ferulic acid, quercetin and rutin (as representative of not-colored phenolics). In this general frame, it is worth mentioning that glycosylated and non-glycosylated anthocyanins are present in almost equivalent amounts in purple wheat [13], whereas most of the non-pigmented phenolics are present in their glycosylated forms. Also, the amount of ferulic acid derivatives in the debranning fractions from pigmented wheat varieties is reportedly much lower than in bran fractions from non-pigmented wheat varieties, where ferulic acid and its glycosylated forms may account for more than 80% of the total phenolics [35].

The results of HPLC profiling carried out on ethanol/HCl extracts from the various samples are reported in Table 3, which also provides—as a reference—the expected content in individual aglycones, calculated from quantitative HPLC profiling of ethanol/HCl extracts from DF. As already observed for the data presented in Table 2, the content of individual anthocyanin aglycones is lower than expected even in the uncooked samples, suggesting that the yield of the extraction procedure may be impaired by processing. This hypothesis is circumstantially supported by the apparent (although not statistically significant) increase in the content of each of the two anthocyanin aglycones in the cooked and lyophilized pasta samples.

A comparison among the data in Table 2; Table 3 also makes it evident that recovery figures for representative anthocyanin aglycones in uncooked pasta (30–50% of the expected, Table 3) are much lower than recovery figures for total anthocyanins (90–65%, Table 2), suggesting that the glycosylated forms of anthocyanins are less sensitive to matrix-related recovery issues. Extending the above comparison to the cooked samples, it appears evident that the abundant glycosylated anthocyanins are much more prone to being released upon cooking than their aglycones, and that anthocyanin aglycones are retained by the matrix in cooked pasta independently of whether the enriched pasta was based on semolina or wheat-flour (see Table 3), at difference with what observed with total anthocyanins (see Table 2).

Figures for the aglycone forms of representative non-pigmented phenolics indicate relevant differences in the efficiency of their incorporation in non-cooked pasta. Whereas ferulic acid was incorporated almost completely regardless of the use of flour or semolina in the formulation, rutin levels in pasta were roughly 30% of what expected, and quercetin fared even worse, showing a 10% incorporation, again regardless of the use of flour or semolina. This is in keeping with the data in Table 2, indicating a 30% retention of total phenolics.

Conversely, bioactive losses upon cooking were highest for the aglycone form of ferulic acid (30–50%) and statistically negligible for the rutin aglycone. In contrast, about 50% of the quercetin aglycone was lost upon cooking flour-based pasta, but no significant losses were observed in the case of semolina-based pasta. In this frame, it should be noted that losses in total phenolics upon cooking were ranging from 50 to 60 percent (see Table 2), again with the lowest losses being recorded for semolina-based pasta.

### 3.3. Inhibition of Enzymes Relevant to Glucose Metabolism and Uptake

Ethanol/HCl extracts from cooked pasta were tested for their capability of inhibiting pancreatic ɑ-amylase and brush border alpha-glucosidase. Cooked pasta was used for these studies, in order to evaluate the possible effects of the bioactives in both types of enriched pasta after undergoing all the required steps prior to their consumption.

**Figure 1:**
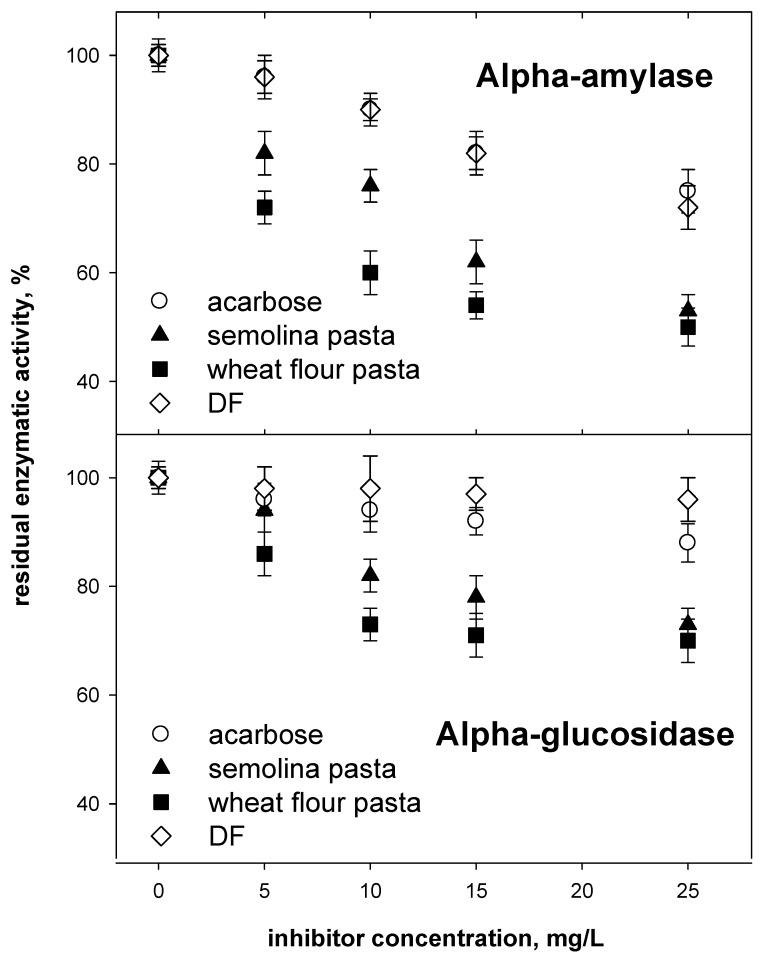
Inhibition of pancreatic alpha-amylase (upper panel) and brush-border alpha-glucosidase (lower panel) by HCl-ethanol extracts from cooked pasta, the original debranning fraction (DF), and by the reference drug, acarbose. Concentration of bioactives in extracts from either type of cooked pasta and from the debranning fraction (DF) is given as cyanidin equivalents. Results are reported as averages ± SD (*n* = 3).

The data in the upper panel of Figure 1 indicate that extracts from either type of pasta had an inhibitory activity towards pancreatic alpha-amylase remarkably higher than the reference drug acarbose or extracts from the debranning fraction (DF) used in the formulation of either type of pasta (on a weight basis, as anthocyanin equivalents). Extracts recovered from semolina-based pasta were found to be less effective inhibitors than those from flour-based pasta, in particular at low anthocyanin concentrations.

A comparison with the profile of individual classes of phenolics in the extracts (see Table 3) does not offer valuable hints for a straightforward interpretation of these results. Further investigation is required to assess whether the difference between extracts from the two pasta samples could depend on the absence of synergistic effects among individual classes of phenolics (and individual chemical species, as reported in previous studies on alpha-amylase inhibition with various classes of phenolics of different origin [11,13,14], possibly as a consequence of losses or alterations of specific individual molecules during the processing and cooking steps.

The data on glucosidase inhibition by extracts from either type of cooked pasta are reported in the lower panel of Figure 1 and indicate that brush border alpha glucosidase is less sensitive to phenolics in the HCl-ethanol extracts (and to the reference drug, acarbose) than pancreatic alpha-amylase, confirming a number of previous reports [13,14]. Also, in the case of alpha-glucosidase, extracts from flour-based pasta appear more active than those from semolina-based pasta at identical concentrations of anthocyanins. As already pointed out for alpha-amylase inhibition, extracts from either type of pasta were much more efficacious in alpha-glucosidase inhibition than extracts from the debranning fraction (DF) used in the formulation of either type of pasta (on a weight basis, as anthocyanin equivalents).

As in the case of alpha-amylase, the differences in alpha-glycosidase inhibitory activity between extracts from the two pasta samples are most evident at low concentrations (<15 mg/L) of bioactives. Again, comparison with data reported in previous work [13] indicates that the inhibitory effects of anthocyanins extracted from cooked pasta samples towards alpha-glycosidase were higher (about 50% inhibition at 25 mg/L total anthocyanin, almost regardless of the type of pasta, see Figure 1) than those for extracts from the same debranning fraction used in this study, which gave approximately 30% inhibition at the same concentrations.

The results discussed above may be interpreted by taking into account the different phenolics profile in the extracts used in this study (see Table 3). These figures suggest that some of the phenolics originally present in DF are better retained in the cooked products than others or, conversely, that the retained ones are more powerful inhibitors than those lost in any of the steps leading to the final cooked pasta. As for the differences observed at low concentrations of anthocyanins in the inhibition assays, a working hypothesis could ascribe them to a different interplay among the individual species that are retained in each system and are responsible for specific inhibition of either enzyme. Indeed, a number of studies have pointed out synergistic inhibitory effects among various molecules in this general class of compounds [10,13,14].

### 3.4. In Vitro Study of Anti-Inflammatory Activity of Cooked Pasta Extracts on Caco-2 Cells

The anti-inflammatory activity of extracts from either type of enriched pasta was tested on the same model used in a number of previous studies [3,4,5]. In short, the model allows us to estimate the effects of extracts for inhibiting IL1β-stimulated synthesis of NF-κB in suitably transfected Caco-2 cells. Anthocyanidins are among the phenolics reportedly able to suppress the expression of inflammatory mediators such as cyclooxygenase (COX-2) by attenuating various forms of cellular signaling, including pathways involving NF-κB and MAPK [1,4,5,6,7,36].

From a methodological standpoint, it has to be underscored that the extracts from cooked pasta samples used in the experiments involving cells were prepared in aqueous ethanol to avoid cell viability issues related to residual traces of HCl [13]. This experimental detail prevents any immediate comparison with the ethanol/HCl extracts discussed above but allows straightforward comparison with the acid-free extracts from the same debranning fraction used in the formulation of the pasta samples used here and already tested for their anti-inflammatory activity in previous studies [13].

**Figure 2:**
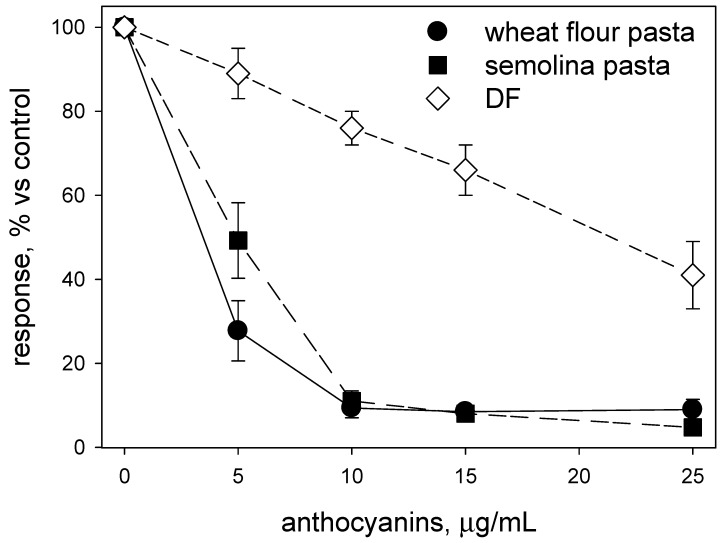
Immunosuppressive effects of the aqueous ethanol extracts from both types of cooked pasta and the original debranning fraction (DF) Data are presented as percent inhibition of IL1β-stimulated expression of NF-κB. Acid-free extracts were used in all these assays. Results are reported as averages ± SD (*n* = 3).

The data in Figure 2 highlight the ability of extracts from both types of cooked pasta to suppress NF-κB expression in the cellular model at concentrations much lower than those required for inhibiting enzymes relevant to glucose metabolism. As observed for enzyme inhibitory activities, the higher efficacy of extracts from flour-based pasta in repressing response to IL-1β with respect to the semolina-based one seems to level off at a high concentration of phenolics (as anthocyanin equivalents).

Figure 2 makes also evident that acid-free extracts from DF were sensibly less efficacious in suppressing NF-κB expression in the cellular model used here than that of similar extracts from either type of cooked pasta all over the concentration of anthocyanins tested here. As discussed above, these differences likely stem from the different phenolics profile in the various extracts (see Table 3). Again, as in the case of enzyme inhibition, some of the phenolics in DF could be better retained in the cooked products than others, and the retained ones may include species that are particularly efficient in suppressing inflammatory response either as individual compounds or through synergistic effects [10,13,14]. Work currently in progress will hopefully contribute to elucidating in sufficient detail the molecular determinants of the observed differences and offer some useful clues as to the suspected synergies among individual components in the extracts.

## 4. Conclusions

Pilot-plant scale incorporation of a phenolics- and fiber-rich fraction (from the debranning of purple wheat) into both semolina- and flour-based pasta was tested at 25% content of the debranning fraction, an amount suitable for avoiding excessive changes in the pasta-making process itself, as well as (1) preserving structural integrity of the product, (2) ensuring some attractive color characters in pasta, and (3) providing added value by incorporating nutritionally relevant amounts of both dietary fibers and phenolics.

Protein solubility and thiol accessibility approaches indicated that protein networks in flour-based enriched pasta were mostly stabilized by hydrophobic interactions and had lower compactness than those in the semolina-based enriched sample. However, a comparison with non-enriched semolina-based pasta indicated that the addition of the bran-derived fraction in the enriched pasta resulted in a sensible destabilization of disulfide-stabilized protein networks in semolina-based systems.

Either type of enriched pasta had a content in anthocyanins and total phenolics much higher than what reported in literature for products obtained from whole pigmented grains, but lower than what was expected from what was calculated from their formulation, at least on the basis of previous analytical data on the debranning fraction of purple wheat used in this study [13,20,21]. Losses in either anthocyanins or phenolics may have occurred as a consequence of thermal treatments in the pasta-making process (most likely as a consequence of the drying steps in pasta production) [7,8,10,15,31]. However, the decrease observed for both anthocyanins and phenolics before cooking could also stem by analytical recovery issues, as suggested by the observation that retention figures in uncooked pasta were lower in semolina-based samples than in flour-based ones.

Cooking induced a further decrease of the phenolics content in both pasta samples. In the case of flour-based enriched pasta, similar cooking losses were observed for anthocyanins alone and total phenolics. Conversely, cooking semolina-based enriched pasta gave a loss in total phenolics slightly lower than those observed in flour-based enriched pasta, but losses in anthocyanins upon cooking semolina-based enriched pasta were the highest, resulting in a final content of these species never exceeding 20% of what was expected on a formulation basis.

HPLC profiling of phenolics was carried out on extracts from either type of enriched pasta both before and after cooking. Although the characterization reported here may hardly be seen as complete, results are suggesting the possible “preferential retention” of specific compounds in each type of enriched pasta, both before and after cooking. The relevance of specific/preferential retention was evident from measurements of biological activities, which were carried out using suitable extracts from cooked samples of either type of enriched pasta.

Extracts from cooked pasta samples were tested for inhibitory capacity towards enzymes involved in glucose metabolism and uptake, and for the ability to suppress the cellular response to inflammatory stimuli. Results of both types of test (i.e., “in vitro” inhibition of enzymes and suppression of response to inflammatory stimuli in a widely used cellular model [1,3,13,36]) indicated that the phenolics retained in both samples of cooked, enriched pasta may be regarded as the “good” ones, at least in terms of biological activity. Indeed, extracts from either type of enriched pasta had inhibitory capacities higher than extracts of the original debranning fraction used for formulating these products at identical concentrations of bioactives.

A coarse estimate from data in Table 2 indicates that a 60 g serving (dry weight) of pasta enriched with suitable fractions from purple wheat debranning could provide between 1.8 and 2.4 mg of total anthocyanins in the cooked product. This should result in an estimated duodenal total concentration of anthocyanins in the range of 4 to 6 mg/L. In this concentration span, the activity of enzymes involved in glucose metabolism is decreased (decrease is in the 20% to 25% range, see Figure 1), as is the response of epithelial cells to inflammatory stimuli (decrease is in the 75% to 60% range, see Figure 2).

The findings reported here may be seen as exciting from the viewpoint of obtaining products with possible physiological impact and of general appeal to the consumer (with the bonus of added nutritional value as a consequence of their content in dietary fibers) by using established production processes. On the other hand, from the standpoint of the food chemist or biochemist, these results bring forward a number of daunting challenges. Indeed, providing a molecular-based rationale for the observations reported above will require substantial efforts, including a more thorough characterization of components in the various samples and, possibly, the use of specific mixtures of individual components to verify, in vitro at least, the observed effects and the occurrence of possible synergies among specific compounds.

## Figures and Tables

**Table 1:** Properties of the protein network in uncooked pasta.

Measured Parameter	Solvent	Enriched Pasta, Wheat Flour	Enriched Pasta, Semolina	Reference Semolina Pasta
soluble proteins, mg/g protein	buffer + NaCl	28.8 ± 1.8 ^a^	21.4 ± 1.6 ^b^	25.0 ± 4.2 ^a^
	+ 8 M urea	90.6 ± 7.2 ^b^	70.1 ± 5.7 ^a^	59.1 ± 9.0 ^a^
	+ 10 mM DTT	129.0 ± 7.8 ^b^	88.5 ± 4.7 ^c^	295.3 ± 29.8 ^a^
accessible thiols, μmol/g protein	buffer + NaCl	0.426 ± 0.060 ^b^	0.213 ± 0.002 ^c^	1.527 ± 0.242 ^a^
	+ 8 M urea	1.320 ± 0.240 ^b^	0.614 ± 0.119 ^c^	6.319 ± 1.346 ^a^

Addition of anthocyanin-rich bran fraction (DF) was considered as a factor for ANOVA. Different letters in a row indicate a significant difference at *p* < 0.05 (Tukey test; *n* = 4). DTT

**Table 2:** Efficiency and stability of phenolics incorporation.

Title	Expected *	Wheat Flour + DF		Semolina + DF	
		Uncooked	Cooked	Uncooked	Cooked
TAC content (mg Cyn-3-O-Glc/kg)	138	122 ± 9 ^a^	51 ± 2 ^c^	89 ± 12 ^b^	31 ± 4 ^d^
TAC retention, % of expected	100	88.4	36.9	64.5	22.4
TAC loss upon cooking, % of uncooked		100	58.2	100	65.2
TPC content (mg GAE/kg)	9480	3110 ± 188 ^a^	1254 ± 98 ^b^	2997 ± 302 ^a^	1467 ± 111 ^b^
TPC retention, % of expected	100	32.8	13.2	31.6	15.5
TPC loss upon cooking, % of uncooked		100	59.7	100	51.1

Addition of DF was considered as a factor for ANOVA. Different letters in a row indicate a significant difference at *p* < 0.05 (Tukey test; *n* = 3). * As calculated from formulation and previous or current analytical data [20].

**Table 3:** Content of representative aglycones in enriched pasta.

Content in Individual Species, μg aglycone/g Pasta		Expected *	Common Wheat Flour + DF		Semolina + DF	
			Uncooked	Cooked	Uncooked	Cooked
Anthocyanins	*Cyanidin*	9.05	3.11 ± 1.10 ^a^	4.89 ± 1.21^a^	4.02 ± 0.81 ^a^	4.92 ± 1.08 ^a^
	*Delphinidin*	13.11	6.07 ± 0.91 ^a^	6.02 ± 1.11 ^a^	6.15 ± 0.81 ^a^	8.78 ± 2.11 ^a^
Phenolics	*Ferulic acid*	4.51	4.10 ± 0.61 ^a^	1.84 ± 0.12 ^c^	4.73 ± 0.39 ^a^	2.38 ± 0.11 ^b^
	*Rutin*	5.00	1.85 ± 0.41 ^a^	1.65 ± 0.08 ^a^	1.73 ± 0.28 ^a^	1.45 ± 0.10 ^a^
	*Quercetin*	4.52	0.48 ± 0.11 ^a^	0.25 ± 0.06 ^b^	0.43 ± 0.07 ^a^	0.38 ± 0.02 ^a^

Different letters in a row indicate a significant difference at *p* < 0.05 (Tukey test; *n* = 4). * As calculated from formulation and previous analytical data on DF [20].
